# Population genetic structure and adaptation of malaria parasites on the edge of endemic distribution

**DOI:** 10.1111/mec.14066

**Published:** 2017-03-15

**Authors:** Craig W. Duffy, Hampate Ba, Samuel Assefa, Ambroise D. Ahouidi, Yacine B. Deh, Abderahmane Tandia, Freja C. M. Kirsebom, Dominic P. Kwiatkowski, David J. Conway

**Affiliations:** ^1^ Department of Pathogen Molecular BiologyLondon School of Hygiene & Tropical MedicineKeppel StLondonWC1E 7HTUK; ^2^ Institut National de Recherche en Sante PubliqueNouakchottMauritania; ^3^ Laboratory of Bacteriology and VirologyLe Dantec HospitalCheikh Anta Diop UniversityDakarSenegal; ^4^ Malaria ProgrammeWellcome Trust Sanger InstituteHinxtonUK

**Keywords:** adaptation, biomedicine, disease biology, ecological genetics, genomics/proteomics, microbial biology

## Abstract

To determine whether the major human malaria parasite *Plasmodium falciparum* exhibits fragmented population structure or local adaptation at the northern limit of its African distribution where the dry Sahel zone meets the Sahara, samples were collected from diverse locations within Mauritania over a range of ~1000 km. Microsatellite genotypes were obtained for 203 clinical infection samples from eight locations, and Illumina paired‐end sequences were obtained to yield high coverage genomewide single nucleotide polymorphism (SNP) data for 65 clinical infection samples from four locations. Most infections contained single parasite genotypes, reflecting low rates of transmission and superinfection locally, in contrast to the situation seen in population samples from countries further south. A minority of infections shared related or identical genotypes locally, indicating some repeated transmission of parasite clones without recombination. This caused some multilocus linkage disequilibrium and local divergence, but aside from the effect of repeated genotypes there was minimal differentiation between locations. Several chromosomal regions had elevated integrated haplotype scores (|iHS|) indicating recent selection, including those containing drug resistance genes. A genomewide *F*_ST_ scan comparison with previous sequence data from an area in West Africa with higher infection endemicity indicates that regional gene flow prevents genetic isolation, but revealed allele frequency differentiation at three drug resistance loci and an erythrocyte invasion ligand gene. Contrast of extended haplotype signatures revealed none to be unique to Mauritania. Discrete foci of infection on the edge of the Sahara are genetically highly connected to the wider continental parasite population, and local elimination would be difficult to achieve without very substantial reduction in malaria throughout the region.

## Introduction

It is important to understand the population genetics of major pathogens, to identify discrete subpopulations that might be controlled, and to study the processes of local adaptation that may be occurring naturally or in response to control efforts. The malaria parasite *Plasmodium falciparum* causes more human deaths and disease than all other eukaryotic pathogens combined, but recent progress in malaria control has led advocacy for elimination from some endemic areas (Newby *et al*. 2016). Genotypic analyses indicate that *P. falciparum* populations have become genetically fragmented in parts of Asia where infection prevalence has been reduced to very low levels (Anderson *et al*. 2000; Anthony *et al*. 2005; Bridle & Vines 2007; Iwagami *et al*. 2009; Pumpaibool *et al*. 2009; Wei *et al*. 2015), and in some parts of Central and South America, this parasite species has become so rare that populations contain very little genetic diversity (Griffing *et al*. 2011; Larranaga *et al*. 2013; Baldeviano *et al*. 2015). However, the potential for *P. falciparum* elimination is much less evident in Africa, the continent with most cases of infection and the highest malaria disease burden. Although the incidence of infection varies throughout different parts of sub‐Saharan Africa, the endemic region is continuous and fragmentation of population genetic structure has not been evident within the continent (Anderson *et al*. 2000; Mobegi *et al*. 2012; Oyebola *et al*. 2014; Bakhiet *et al*. 2015).

Malaria parasites are dependent upon a human host and mosquito vector for survival, the latter of which is itself reliant upon open bodies of fresh water for larval development, so malaria transmission across Africa is closely linked to rainfall (Gething *et al*. 2011). Malaria remains highly prevalent in most of West Africa, but infection incidence has recently decreased in The Gambia and Senegal where malaria prevention and treatment have become more widely used (Ceesay *et al*. 2008, 2010; Trape *et al*. 2012; Daniels *et al*. 2015). To the north of these countries the limit of the malaria‐endemic region occurs in Mauritania, on the edge of the Sahara desert (Gething *et al*. 2011; Lekwiry *et al*. 2015). To establish whether *P. falciparum* could be eliminated from any part of the region, it is particularly important to evaluate the feasibility in Mauritania, where transmission is mainly due to the mosquito vector *Anopheles arabiensis* that can survive in arid environments with limited seasonal annual rainfall (Dia *et al*. 2009). A small study involving genotyping of parasites with three polymorphic markers suggested unstable malaria transmission in one town in southern Mauritania, as a clonal outbreak occurred after a period of drought, presumably due to single genotype infections in human cases and self‐fertilization of parasites in the mosquito vectors (Jordan *et al*. 2001). Detailed studies are needed of the population genetics of malaria in this important part of the edge of the endemic distribution, including approaches to identify whether there is evidence of local positive selection. As there are more than 5000 genes in total in the ~23 megabase (Mb) parasite genome with 14 chromosomes, multilocus and ideally genomewide analyses are required.

To thoroughly investigate the parasite population genetics in this extreme environment on the edge of the species range, multilocus microsatellite genotyping was first performed to examine the population structure of *P. falciparum* at multiple locations across its distribution in Mauritania. Following this, Illumina short read sequencing was performed on further samples to enable a genomewide scan for signatures of selection. This allowed testing of the hypothesis that a low transmission environment and distantly separated human settlements would limit parasite gene flow and give rise to genetically isolated parasite populations with low levels of diversity. Further, it enabled testing of a hypothesis that adaptation to a low transmission environment may occur, potentially affecting loci regulating production of the transmission‐stage gametocytes which are required to infect mosquitoes during a very short period each year.

## Materials and Methods

### Study populations and sample collection

**Figure 1:**
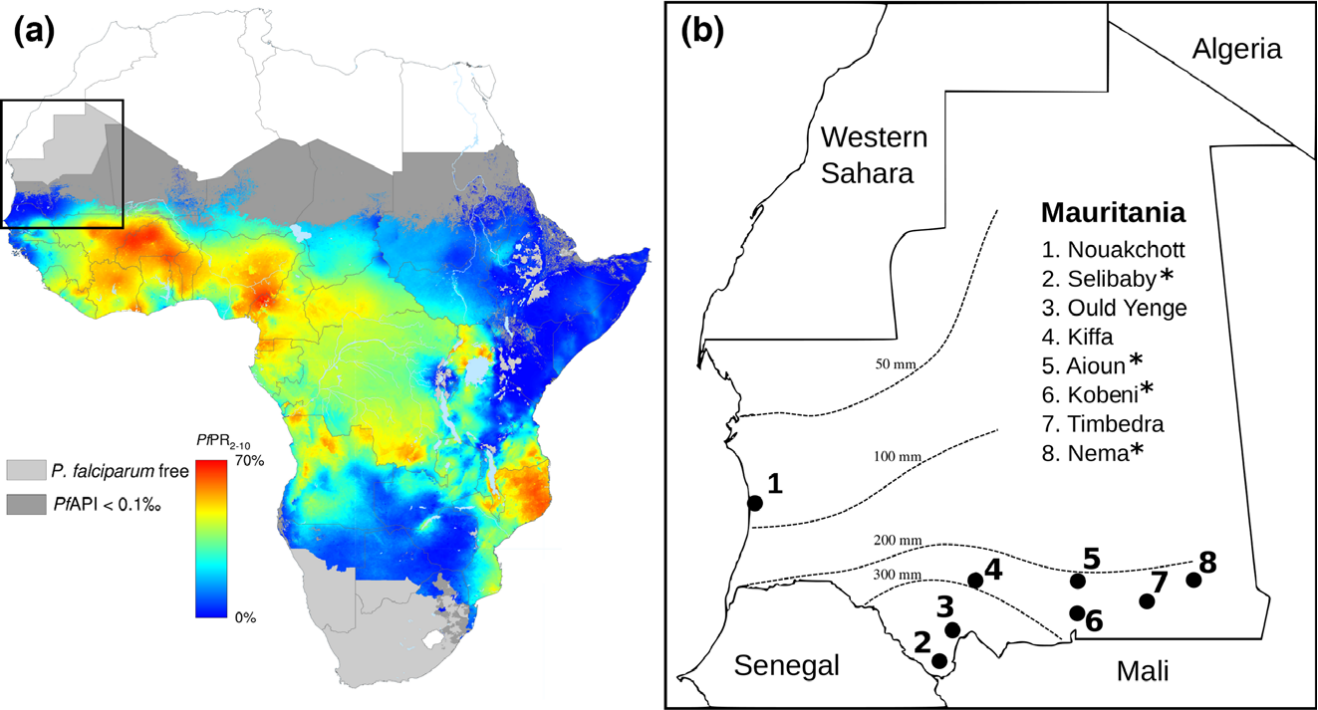
Map showing locations of sampling sites in Mauritania. (a) Map of *Plasmodium falciparum* distribution in Africa (Gething *et al*. 2011), with a rectangle showing the area covered in the enlarged map of Mauritania on the edge of the endemic distribution. The heatmap shading indicates estimated prevalence of *P. falciparum* infection in children between 2 and 10 years of age throughout its endemic range, and grey shading shows areas where the parasite is extremely rare or absent. (b) Locations of eight malaria‐endemic sites across Mauritania from which clinical samples were collected for *P. falciparum* genotypic analysis. Multilocus microsatellite genotype data were generated on parasites from 203 patients with malaria sampled across all eight sites in 2012 or 2013, with sample sizes for each site being shown in Table 1. Whole‐genome sequence data were subsequently generated on parasites from another 86 patients with malaria sampled in 2014 from four of the sites, marked with asterisks (*). The dashed lines indicate isohyets of annual rainfall which occurs in a short season, mostly between July and September.

The large country of Mauritania contains three major ecological zones, with the Sahara desert in the northern two‐thirds of the territory, a band of Sahel spanning the country to the south of the Sahara, and a narrow river valley zone along the border with Senegal in the southeast. Malaria caused by *Plasmodium falciparum* occurs in the latter two zones in the south of the country, at the extreme edge of its continental distribution (Fig. 1a) (Ba *et al*. 2016).

First, to survey local population genetic structure of *P. falciparum* by microsatellite genotype analysis, blood samples were collected from patients attending local health facilities at eight different geographical sites in the country during two annual malaria seasons, between August and December in 2012 and 2013 (Fig. 1b). Samples were collected from one site (Nouakchott) in both years and from each other site in one of the years (the Kobeni, Aioun and Timbedra sites in 2012; the Selibaby, Ould Yenge, Kiffa and Nema sites in 2013). Malaria was diagnosed by local health facility staff using rapid diagnostic tests, and patients with positive results were invited to provide finger prick blood samples, collected on filter papers for subsequent DNA extraction using QIAmp DNA minikits. All samples analysed were collected from local residents who reported that they had not travelled during the past 2 weeks. These samples were tested for the presence of different malaria parasite species by species‐specific PCR as previously described (Ba *et al*. 2016), and 203 of those positive for *P. falciparum* were genotyped at a panel of 10 microsatellite loci as described below.

Subsequent sampling during the 2014 malaria season was undertaken, to survey genomewide sequence polymorphism and enable analysis of loci under selection. Venous blood samples were collected from patients presenting with malaria at four of the previously sampled sites (Aioun, Kobeni, Selibaby and Nema; Fig. 1b), and these were leukocyte depleted immediately following collection using CF11 cellulose powder filtration columns (Venkatesan *et al*. 2012) prior to being frozen at −20 °C. DNA was extracted from frozen samples using the QIAamp blood midi kit, and for 86 of the samples, the quality and purity of *P. falciparum* DNA was sufficient to allow processing for whole‐genome paired‐end short read sequencing on an Illumina HiSeq.

Ethical approval for the study was provided by the ethics committees of the Ministry of Health in Mauritania and the London School of Hygiene and Tropical Medicine. Samples were collected after written informed consent from patients, or the guardians of patients who were under 18 years of age.

### Microsatellite genotyping and population genetic structure

Parasite DNA from each of 203 *P. falciparum*‐positive fingerprick blood samples collected in 2012–2013 was genotyped with a set of 10 highly polymorphic microsatellite markers, following an established hemi‐nested PCR protocol (Anderson *et al*. 1999) with a modified combination of fluorescent dye labels on internal primers (Mobegi *et al*. 2012). The PCR product sizes were determined by electrophoresis on an ABI 3730 Genetic Analyzer, and after visual inspection to ensure quality, these were scored using Peak Scanner 2 software, with multiple alleles called if any additional allele had a peak height of at least 25% that of the major allele in the infection. A conservative count of the number of genotypes within each infection, termed the multiplicity of infection (MOI), was defined as the highest number of alleles observed at any individual locus within the individual. For all other population genetic analyses using the microsatellite data, only the major allele at each locus within each infection was counted. The presence of multilocus linkage disequilibrium was tested by calculation of the standardized index of association $I_{A}^{S}$ using lian 3.0 (Haubold & Hudson 2000) with significance testing by 10000 iterations of Monte Carlo random sampling. Pairwise fixation indices (*F*_ST_, based on the Ɵ coefficient) and significance values between populations with at least 10 isolates were calculated using fstat version 2.9.3.2, updated from (Goudet 1995), with *F*_ST_ averaged across the 10 genotyped loci being taken as an appropriate unbiased estimator of divergence (Balloux & Lugon‐Moulin 2002). Potential association between *F*_ST_ and geographical distance was explored by a Mantel test of matrix correlation using genepop 4.0.10 (Rousset 2008). An additional measure of differentiation (Jost's *D*_est_) was calculated in genalex 6.501 (Peakall & Smouse 2012) using all samples. Population substructuring was assessed using PCA plots calculated in Genalex 6.501, and structure analysis was run 10 times using structure 2.3.4 (Pritchard *et al*. 2000; Falush *et al*. 2003; Hubisz *et al*. 2009) with an admixture model for K 1‐10, 20 000 MCMC reps with a burn‐in of 10 000 reps. We estimated the effective population sizes for each season under both a stepwise‐mutation model and an infinite allele model as previously described (Anderson *et al*. 2000), with an estimated microsatellite mutation rate of 1.59 × 10^−4^ (95% confidence intervals: 6.98 × 10^−5^–3.70 × 10^−4^) (Jennison *et al*. 2015).

### *Plasmodium falciparum* genome sequencing and population genomic analyses

Parasite DNA prepared from the clinical infections sampled in 2014 were processed for whole‐genome paired‐end short read sequencing on an Illumina HiSeq following the pipeline for quality control and sample preparation at the Wellcome Trust Sanger Institute. Reads were aligned to the *P. falciparum* 3D7 v3 reference genome and SNPs called (Manske *et al*. 2012) as performed for the malariagen 5.1 data from other populations. High‐quality SNPs were defined as those that passed all VCF filters or only failed the ‘Coding Type’ filter (allowing for retention of intergenic SNP positions). Genotype calls were made for each infection sample at all SNPs covered by a minimum of 10 reads. The data set was filtered iteratively by alternatively excluding isolates and SNPs in a stepwise manner. During the first iteration, isolates with >90% missing data were excluded prior exclusion of SNPs with >90% missing data. The percentage of missing data was recalculated following each removal of isolates or SNPs. The level of missing data allowed was decreased in steps of 5%, with multiple steps per iteration if all isolates or SNPs were below the threshold. The process was repeated until all isolates and all SNPs had less than 5% missing data, with 65 of the initial 86 isolates passing this filtering process and being used for subsequent analysis.

Within‐infection genomic diversity was assessed using the *F*_WS_ fixation index, estimating on a scale from 0 to 1 the fixation of alleles within each infection sample relative to the diversity observed in the total population sample (Auburn *et al*. 2012; Manske *et al*. 2012). Isolates with *F*_WS_ indices >0.95 tend to have a single predominant genotype while those with lower indices are clearly mixed genotype infections. The *F*_WS_ index values were calculated as previously described using custom R and Perl scripts to calculate within host allele frequencies from per isolate VCF files. The relationships between isolates were determined using a pairwise similarity matrix and visualized with an unrooted neighbour‐joining tree using the Ape package for R, or through calculation of principal components. Population structuring was assessed using admixture 1.3.0 (Alexander *et al*. 2009) for all SNPs with a minor allele frequency >0.05 (10 duplicate runs for K 1‐10, 10‐fold cross‐validation and standard error estimation with 1000 bootstraps). Weir and Cockerham's *F*_ST_ and Jost's *D* were estimated for each SNP using the diversity package for R (Keenan *et al*. 2013).

Tajima's *D* values were calculated to assess the allele frequency spectrum for each gene with at least 3 SNPs using custom R scripts as applied to previous population samples (Duffy *et al*. 2015). As there were missing SNP data in subsets of isolates at each gene, isolates were excluded on a per gene basis to retain those with complete data for the analysis of each gene separately. Signatures of directional selection within Mauritania were identified using the standardized integrated haplotype score (|iHS|) statistic for each SNP with a minor allele frequency of >0.05 (Voight *et al*. 2006; Gautier & Vitalis 2012), while an *Rsb* scan (Tang *et al*. 2007) for population‐specific selection was performed by comparing the rate of haplotype decay in the Mauritanian population with the rate of decay in a previously published West African population sample from a highly endemic area in the Republic of Guinea (Mobegi *et al*. 2014). The |iHS| and *Rsb* analyses were performed using the rehh package for R (Gautier & Vitalis 2012) using SNPs with a minor allele frequency of >5% and unique isolates only. The ancestral *P. falciparum* allele was determined by alignment with the *Plasmodium reichenowi* genome, with positions discarded if an ancestral allele could not be determined (Otto *et al*. 2014). Recombination maps were estimated from the mean of 5 independent runs of LDhat with a block penalty of 20, 10 million rjMCMC iterations and a burn in of 100 000 iterations (Auton & McVean 2007). Recombination parameters across a region were calculated, on the basis of the median estimated within each sliding window of 21 SNPs. Putative genomic regions under local selection were identified as those with multiple SNPs having |iHS| values >3.29 (top 0.1% of genomewide values), and at least one SNP with a value >5. Windows were defined by calculating the distance required for the linkage disequilibrium of extended haplotypes around these SNPs to decay to 0.05 of maximal possible values, with overlapping windows combined into continuous windows (windows with only a single high scoring SNP were discarded). In the *Rsb* scan, windows with multiple SNPs having absolute *Rsb* values >5 (with a positive or negative sign) were taken to indicate loci most likely to be under local selection.

## Results

### *Plasmodium falciparum* population structure in Mauritania analysed using microsatellites

Multilocus microsatellite genotypes were successfully obtained from 203 *Plasmodium falciparum*‐positive malaria cases which had been sampled from eight diverse sites in Mauritania during the 2012 and 2013 transmission seasons (Fig. 1). Complete 10‐locus genotypes were obtained for 179 of these infections, with at least 7 loci being scored for each of the remainder (Table S1, Supporting information). The numbers of different alleles observed per locus ranged from 7 to 18, and the allelic diversity (*H*_e_) per locus ranged from 0.39 to 0.89 (Table S2, Supporting information). There was no significant variation in allelic diversity among different sites in the country, or between the 2012 and 2013 seasons, with mean *H*_e_ values across all 10 loci being between 0.73 and 0.77 at each site. As expected from the similar distributions of allelic diversity, estimates of effective population size based on an average microsatellite mutation rate were similar for all of the sampled local populations in Mauritania (Table S3, Supporting information). In all cases, the estimated values were higher under a stepwise‐mutation model (point estimates ranging from 9195 to 16 954) than under an infinite alleles model (point estimates ranging from 4023 to 5885), as reported previously for analyses of other endemic *P. falciparum* populations (Anderson *et al*. 2000).

**Figure 2:**
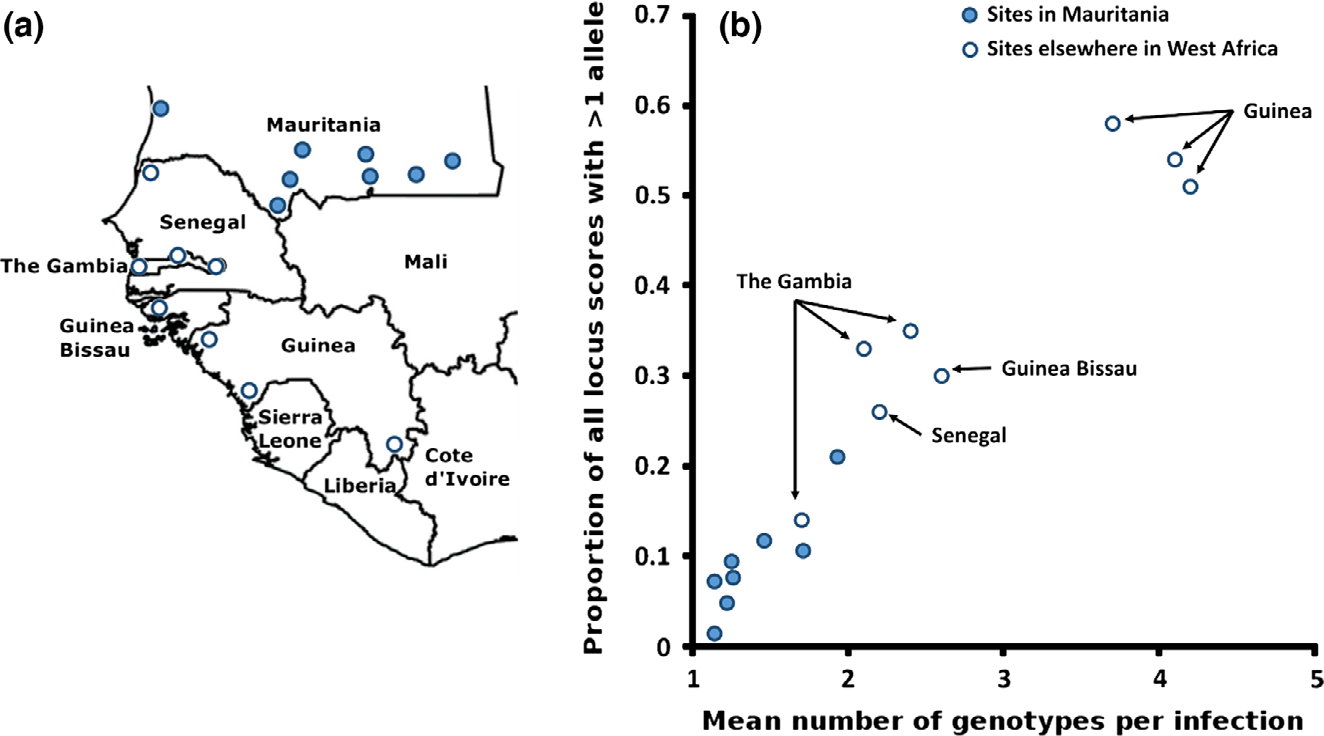
Sites in Mauritania have less genotypically mixed *Plasmodium falciparum* infections than elsewhere in West Africa. (a) Locations of eight sites sampled in Mauritania, and eight in other West African countries to the south (in Senegal, The Gambia, Guinea Bissau and Republic of Guinea). The eight sites sampled in Mauritania from which infections were genotyped for a panel of ten microsatellite loci are as described in Fig. 1 and Table 1, whereas details for the eight other West African sites analysed with the same set of microsatellite loci are previously published (Mobegi *et al*. 2012). (b) Two different indices are plotted, each showing significantly lower genotypic complexity of infections at sites in Mauritania than elsewhere (Mann–Whitney test, *P* < 0.001 for each index). The only non‐Mauritanian site with unusually low levels of mixed genotype infections, within the range of values seen in Mauritania, is a major urban area on the Atlantic coast of The Gambia where malaria infection endemicity is known to be lower than elsewhere (Ceesay *et al*. 2010).

The numbers of different parasite genotypes detected per clinical infection in Mauritania were low (Table 1). At six of the sampled sites, the majority of infections contained only a single genotype, while in Aioun and Kobeni, there were more infections containing two or more genotypes (*P* = 0.002 and *P* < 10^−7^ for comparisons of these respective sites with the other sites combined). Overall, the mean number of genotypes detected per infection was 1.56 and the proportion of all locus scores that had more than one allele was 0.13. As predicted from the low infection endemicity locally, the sites sampled in Mauritania had lower proportions of mixed genotype infections than seen elsewhere in West Africa (Fig. 2).

**Table 1:** Levels of genotypic mixedness of *Plasmodium falciparum* clinical infections sampled from eight diverse sites in Mauritania and genotyped for a panel of ten microsatellite loci

Location	Number of infections genotyped	Number of infections with each of the following numbers of different genotypes detected				Mean number of genotypes per infection	Proportion of locus scores with >1 allele
		1	2	3	4		
Nouakchott	23	17 (74%)	6 (26%)	0	0	1.26	0.076
Selibaby	23	19 (83%)	3 (13%)	1 (4%)	0	1.22	0.048
Ould Yenge	13	9 (69%)	2 (15%)	2 (15%)	0	1.46	0.117
Kiffa	7	6 (86%)	1 (14%)	0	0	1.14	0.014
Aioun	17	7 (42%)	8 (47%)	2 (12%)	0	1.71	0.106
Kobeni	83	26 (31%)	42 (51%)	10 (12%)	5 (6%)	1.93	0.210
Timbedra	16	13 (81%)	2 (13%)	1 (6%)	0	1.25	0.094
Nema	21	18 (86%)	3 (14%)	0	0	1.14	0.072

**Figure 3:**
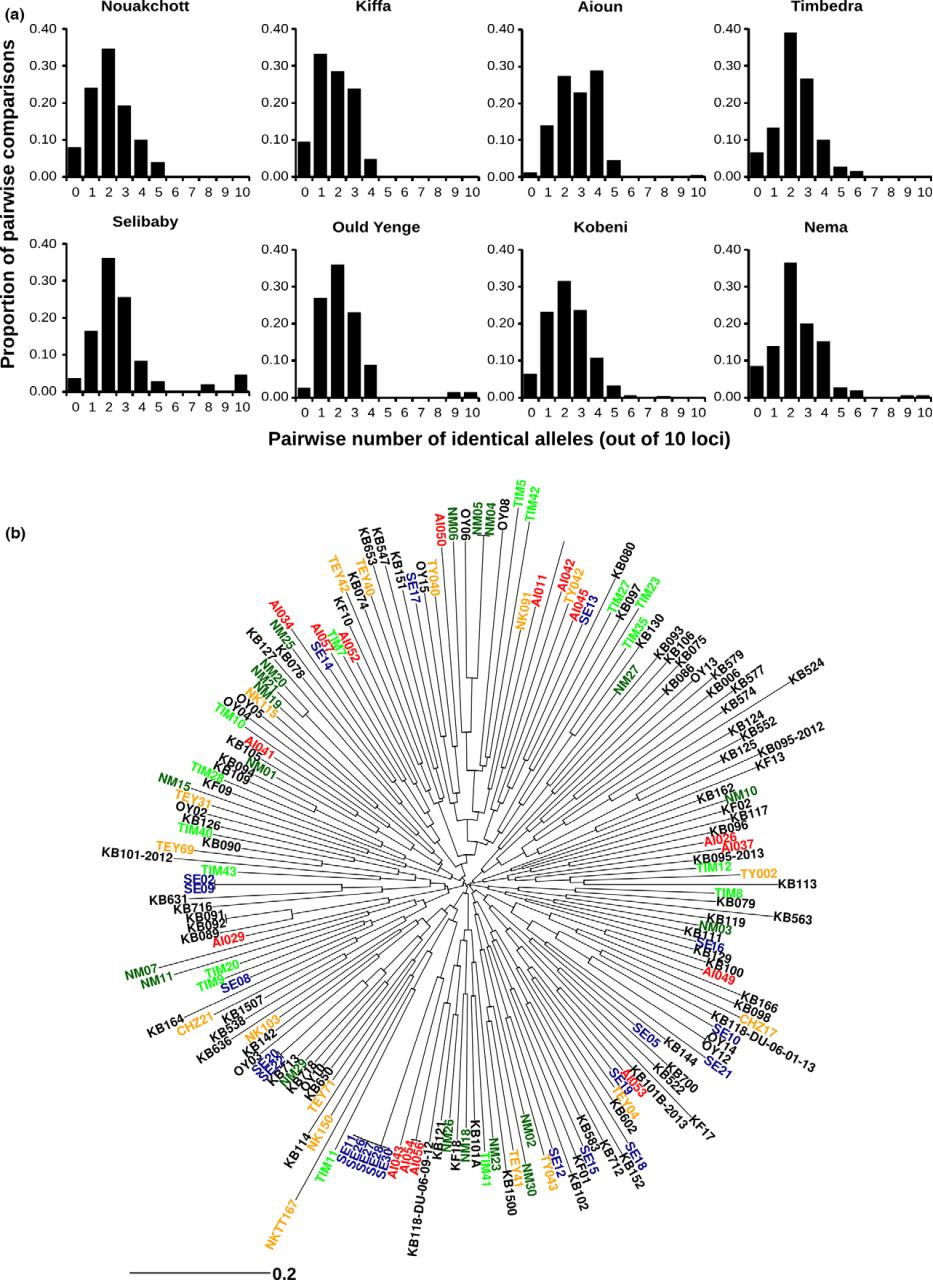
Pairwise similarity of *Plasmodium falciparum* microsatellite genotypes among different clinical infections within each of the sampled sites in Mauritania. For each infection, the predominant allele at each of the panel of 10 loci was considered for the genotypic profile, ignoring minority alleles in the case of mixed genotype infections. (a) Numbers of identical alleles in pairwise comparisons, showing that most infections differ from each other at most loci, with a small number of exceptions where pairs of infections were closely similar. (b) Neighbour‐Joining dendrogram showing low levels of similarity of the 10‐locus parasite genotypes between most infections, contrasted with small local clusters of closely related or identical genotypes. Infections from each of the eight sampling sites are shown in a different colour (blue indicates those from Selibaby where there was the highest proportion of related genotypes).

Pairwise comparison of infections with complete 10‐locus microsatellite genotype profiles showed that most of them were unrelated, having identical alleles at only 2 or 3 loci on average (Fig. 3). However, against this background there was a minority of highly related pairs, matching for at least 7 of 10 loci. The majority of these (23 of 37 related pairs, 62.2%) were from the same local population, in Kobeni, Aioun, Nema, Ould Yenge or Selibaby. Notably, 9 of the 22 infections in Selibaby were genotypically identical to at least one other infection locally, and one genotype was seen in five different infections. The occurrence of highly related genotypes gave rise to significant multilocus linkage disequilibrium, as assessed by the presence of significant local values of the index of association in four of the populations. When identical genotypes were counted only once in analysis, the statistical significance disappeared in all except one of the populations (Table 2).

**Table 2:** Index of association ($I_{A}^{S}$) testing for multilocus linkage disequilibrium in local populations of *Plasmodium falciparum* sampled from each of eight diverse sites in Mauritania and genotyped at ten microsatellite loci widely separated in the genome

Location	Including all isolates			Unique genotypes		
	*N*	$I_{A}^{S}$		*N*	$I_{A}^{S}$	
Aioun	16	0.039	**	15	0.002	NS
Kiffa	7	0.000	NS	7	0.000	NS
Kobeni	69	0.006	NS	62	0.003	NS
Nema	20	0.048	**	17	0.028	*
Nouakchott	18	0.000	NS	18	0.000	NS
Ould Yenge	11	0.038	*	10	0.000	NS
Selibaby	22	0.196	***	16	0.000	NS
Timbedra	16	0.007	NS	16	0.007	NS

Tests of the null hypothesis $I_{A}^{S}$ = 0: *, *P* < 0.05; **, *P* < 0.01; ***, *P* = 0.001; NS, not significant (*P* > 0.05).

**Figure 4:**
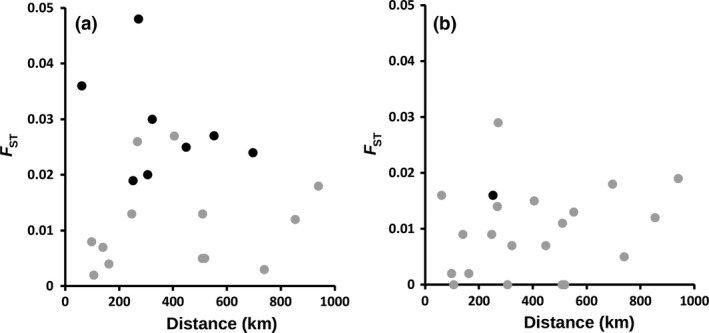
Scatterplot of *F*_ST_ genetic differentiation in all pairwise comparisons of eight local Mauritanian *Plasmodium falciparum* populations sampled vs. the geographical distance between them. (a) *F*_ST_ values calculated with the inclusion of all 203 infection samples, and (b) *F*_ST_ values calculated with 182 samples following removal of near identical genotypes within sites (randomly retaining one of the samples matching at 7 or more of 10 loci). Although significant *F*_ST_ values (black points show uncorrected *P* values of <0.01) were observed for 8 pairs of sites when all isolates were considered, only one (comparing Aioun and Nema) remained following removal of replicate near identical infection samples. All *F*_ST_ values for all pairs of sites, as well as values of another differentiation index (Jost's *D*_est_), are listed in Table S4 (Supporting information).

Principal coordinate analysis (PCoA) of the multilocus genotypes of each isolate did not show any separate clustering of samples from the different sites, or from the two different years (Fig. S1, Supporting information). Clustering analysis using structure 2.3.4 was unable to distinguish the individual sites or sampling years under an admixture model including prior sample group information. Comparisons of allele frequencies among seven of the sites (excluding the population sample from Kiffa that had a very small sample size) identified low but significant differences (*P* < 0.05) in eight of 21 pairwise comparisons, with *F*_ST_ values ranging up to 0.048. Two particular sites (Selibaby and Aioun) were involved in each of the comparisons that showed significant differences (Table S4, Supporting information), and there was no significant correlation between *F*_ST_ values and the geographical distance between sites overall (Fig. 4a). Exclusion of closely related infection genotypes from the analysis markedly reduced the differences between sites, indicating the effect of local expansion of related genotypes on the population structure (Fig. 4b; Table S4, Supporting information).

### Genomewide analysis of *Plasmodium falciparum* population structure and signatures of selection in Mauritania

**Figure 5:**
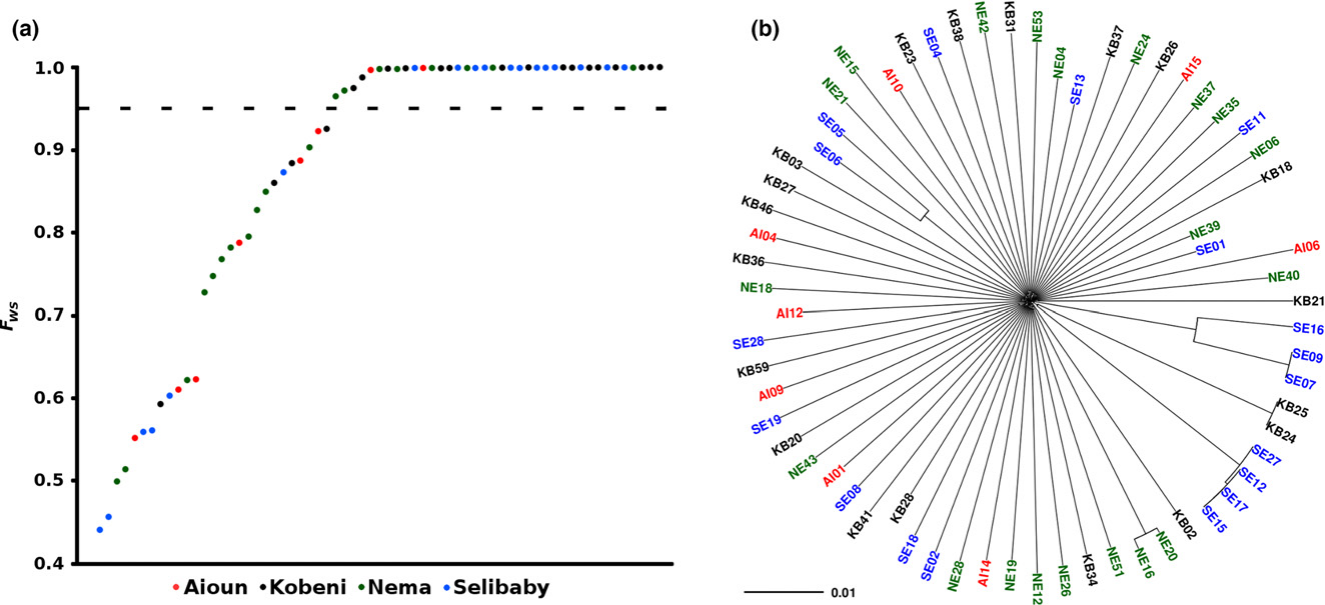
Genomewide sequence analysis of *Plasmodium falciparum* diversity within and among 65 different infections sampled from Mauritania. The isolates are coloured according to sampling location (red, Aioun; black, Kobeni; blue, Selibaby; green, Nema). (a) Within‐infection fixation indices (*F*_WS_) of individual samples show that most are dominated by single genotypes, having *F*_WS_ values approaching 1.0 (the dashed line indicates the Fws value of 0.95). (b) Neighbour‐Joining tree based on a distance matrix of pairwise SNP identity shows that most of the infections are unrelated and only a minority have similar genotypes. The pairwise distances were calculated using 45 472 biallelic SNPs, and the scale bar indicates the length of a branch corresponding to difference at 1% of SNP positions. Highly related isolates were identified here, and only one of each type was retained for subsequent genomewide analyses to scan for loci with extended haplotypes (isolates excluded were KB24, NE16, SE06, SE07, SE09, SE12, SE15 and SE17). Individual sample information and Accession nos are available on a dedicated project page https://www.malariagen.net/resource/22 and in Table S5 (Supporting information).

Genomewide sequence data were obtained from 86 clinical *P. falciparum* infections from 4 sites in Mauritania that were sampled in 2014, with 65 of these being selected for population genomic analyses as they had <5% missing SNP coverage (samples from Nema *n* = 20, Kobeni *n* = 19, Selibaby *n* = 18 and Aioun *n* = 8) (Table S5, Supporting information). Mapping of paired‐end reads to the 3D7 reference genome and SNP calling using a stringent pipeline identified 45 472 biallelic SNPs among the 65 infection samples with high coverage, of which 10 371 SNPs (22.8%) had an overall minor allele frequency of at least 5%. Analysis of the within‐infection fixation index *F*_WS_ shows that only a minority of the infection samples had low values that indicate mixed genotypes (Fig. 5a). The average *F*_WS_ value across all infections was 0.87, which is higher (indicating less mixed infections) than seen previously in comparable sequence data from highly endemic African population samples (Auburn *et al*. 2012; Mobegi *et al*. 2014; Duffy *et al*. 2015).

As with the microsatellite analysis of isolates from the previous 2 years, the whole‐genome sequence data from different sites sampled within Mauritania did not show separate clusters using PCA (Fig. S2, Supporting information), and admixture analysis also indicated support for a single population (*K* = 1, cross‐validation error 1.17). The majority of the genomewide SNP profiles from different infections were unrelated, but there was a small number of infections that had closely related genotypes (Fig. 5b). This was most notable in infections from Selibably, consistent with the results obtained by microsatellite analysis of samples from the previous years. Genomewide average *F*_ST_ values between the sampling locations were low (considering locations with more than 10 isolates sequenced, *F*_ST_ values for Selibaby vs. Kobeni, 0.013; Selibaby vs. Nema, 0.015; Kobeni vs. Nema 0.003) and were not significantly different from zero. Consistent with the results from the microsatellite analyses of samples from the previous years, this is consistent with high levels of gene flow within the region, so the isolates were considered as a single population for subsequent analysis. The overall allele frequency distribution for the Mauritanian population was negatively skewed, with a mean Tajima's *D* value of −1.67, averaged over 2965 genes that each had at least 3 SNPs (Table S6, Supporting information). This summary of the allele frequency spectrum is similar to that seen in other African populations, and consistent with historical population expansion of *P. falciparum*.

**Figure 6:**
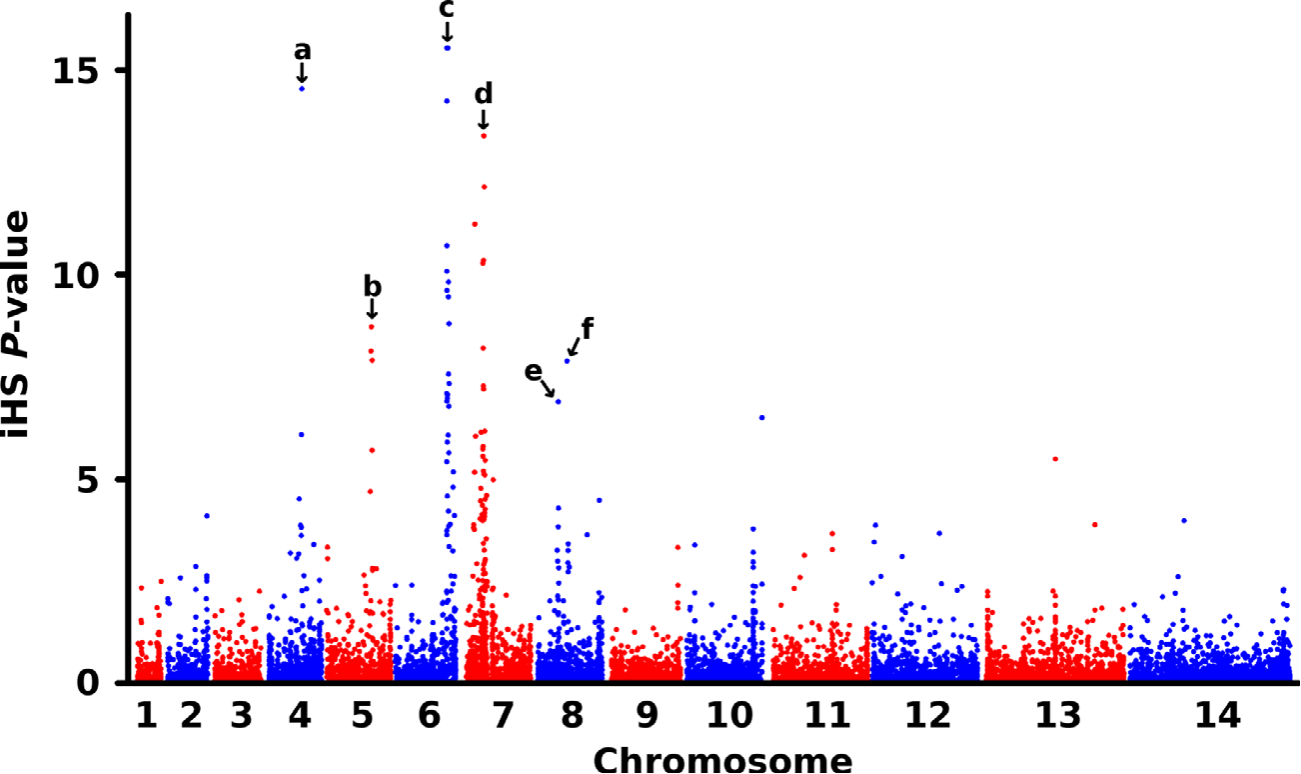
Genomewide scan for evidence of loci under positive directional selection using the standardized integrated haplotype score, plotted as ‐log10 (*P*‐value). Scores were calculated for 10 371 SNPs with minor allele frequency >5% using 57 unique Mauritanian clinical isolate sequences after random removal of isolates sharing >96% SNP identity with any other isolate. The scan identified 6 regions of the genome (labelled a–f) with strongest evidence of extended haplotypes: (a) chromosome (chr) 4 map region 673–765 kb covering 23 genes from PF3D7_0415200 to PF3D7_0417400, (b) chr 5 map region 908–1000 kb covering 17 genes from PF3D7_0522400 to PF3D7_0524000, (c) chr 6 map region 1087–1271 kb covering 33 genes from PF3D7_0627100 to PF3D7_0630300, (d) chr 7 map region 196–701 kb covering 119 genes from PF3D7_0704300 toPF3D7_0715900, (e) chr 8 map region 486–506 kb covering 3 genes from PF3D7_0809600 toPF3D7_0809800, (f) chr 8 map region 626–703 kb covering 21 genes from PF3D7_0812500 to PF3D7_0814500 (Table S7, Supporting information). Three of the six regions, on chr 4, 5 and 7, include antimalarial drug resistance genes *dhfr*,* mdr1* and *crt,* respectively, while the two regions on chromosome 8 are positioned to either side of the drug resistance gene *dhps*. Only the region on chromosome 6 is not associated with a known drug resistance gene.

To scan for evidence of loci under recent directional selection, the standardized integrated haplotype score (|iHS|) was calculated for all SNPs with minor allele frequencies >5% in the overall Mauritanian population sample. This test identifies chromosomal regions which are likely to have been subject to recent positive selection, by identifying alleles associated with extended haplotypes relative to the alternate allele at that position. The short generation time and high recombination rate in malaria parasites quickly breaks down these haplotypes, so signatures of elevated |iHS| are generally indicative of recent selection upon the parasite population. The results identified six regions where elevated |iHS| values were associated with three or more SNPs (Fig. 6 and Table S7, Supporting information). The genomic windows showing the strongest evidence of selection were on chromosome 7 (incorporating the locus encoding chloroquine resistance transporter, *crt*), and towards the end of chromosome 6. Additional windows of elevated |iHS| values were observed in regions of chromosomes 4 and 5 that, respectively, included the antimalarial drug resistance genes *dhfr* (encoding the antifolate drug target dihydrofolate reductase) and *mdr1* (encoding the multidrug resistance 1 transporter). The antimalarial resistance gene *dhps* (encoding the antifolate drug target dihydropteroate synthase) is situated between the two closely adjacent windows of elevated |iHS| values on chromosome 8.

### Genomewide comparisons of *Plasmodium falciparum* in Mauritania with a population sample from a more highly endemic area in West Africa

**Figure 7:**
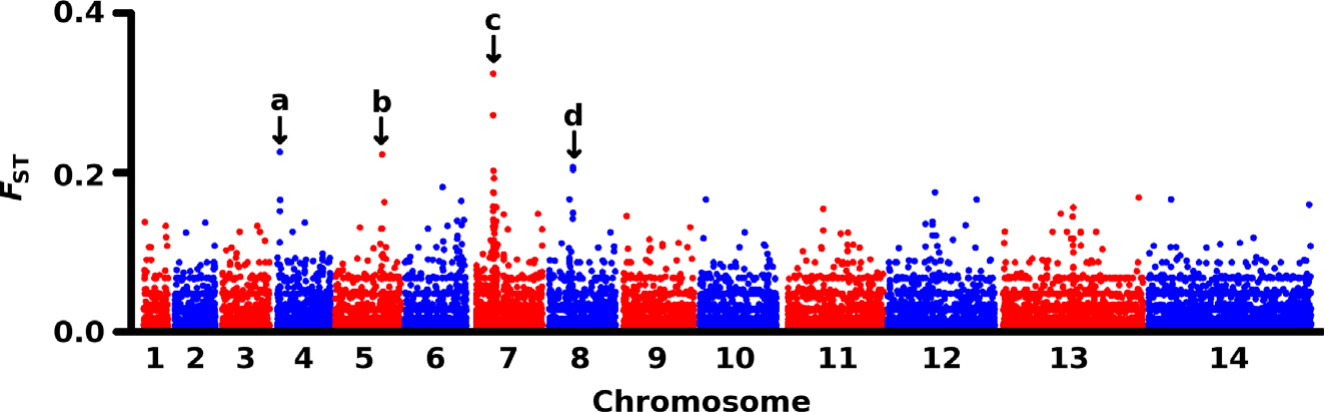
Scan for allele frequency divergence across the *Plasmodium falciparum* genome between Mauritania (65 clinical isolates) and a more highly endemic population in Guinea (105 clinical isolates), as measured by *F*_ST_ with 69 913 SNPs genomewide. The mean genomewide *F*_ST_ was 0.004, but nine SNPs had *F*_ST_ values >0.2, clustered in 4 loci (labelled a to d) on chromosomes 4, 5, 7 and 8: (a) one SNP on chromosome (chr) 4 within the *Rh1* gene (PF3D7_0402300; SNP position 138308, nonsynonymous E191K), (b) one SNP on chr 5 located 4.1 kb from the drug resistance gene *mdr1*, (c) 5 SNPs on chr 7 located 2.8–8.9 kb from the drug resistance gene *crt*, (d) two SNPs 3.7 kb apart on chr 8 with one in the drug resistance gene *dhps* (PF3D7_0810800; SNP position 549 685, nonsynonymous codon G437A having a known role in conferring resistance to sulfadoxine).

The genomic regions indicated to have been under selection above have also been highlighted from scans for evidence of recent directional selection in other *P. falciparum* populations, including a large population sample from a highly endemic area ~1000 km to the south of Mauritania, in the forested region of the Republic of Guinea where malaria transmission occurs throughout most of each year (Mobegi *et al*. 2014). Sequence data from 105 Guinean clinical infections were compared with the overall sample of 65 infections from Mauritania, with a total of 69 913 SNPs across the two populations. This shows that the population samples do not separate into different clusters by PCA (Fig. S3, Supporting information), while ADMIXTURE analysis was also unable to separate the two populations (with the best support for *K* = 1, cross‐validation error 0.44). The genomewide mean *F*_ST_ between the samples from these different countries was only 0.004, but there were four genomic loci at which SNPs had *F*_ST_ values above 0.2 (Fig. 7). Three of these were in or adjacent to antimalarial drug resistance genes (on chromosome 5, one SNP 4 kb away from the *mdr1* gene; on chromosome 7, five SNPs closely situated to the *crt* gene, the nearest being 2.8 kb away; on chromosome 8, two closely situated SNPs with one being in the *dhps* gene; Table S8, Supporting information). Aside from these drug resistance loci, the only SNP with *F*_ST_ > 0.2 was in the *Rh1* gene on chromosome 4 which encodes a ligand expressed by merozoite stage parasites to enable invasion of erythrocytes (Table S8, Supporting information). Genomewide scan of the SNP data with another index to compare allele frequencies (Jost's *D*_est_) also showed these loci to be highly differentiated (Fig. S4, Supporting information).

**Figure 8:**
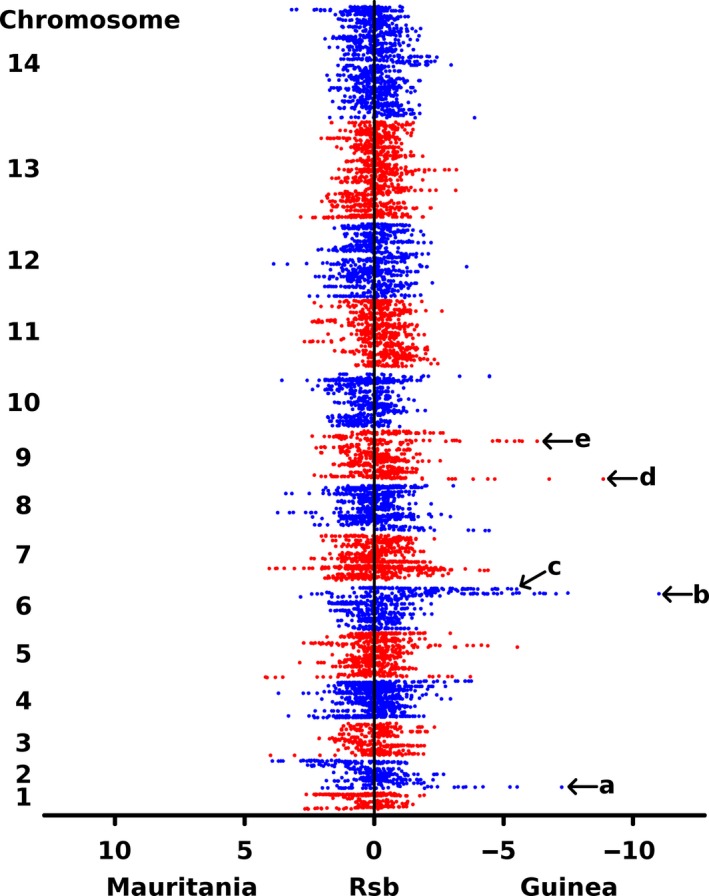
Genomewide scan for evidence of population‐specific directional selection using the *Rsb* metric comparing Mauritania to an area in West Africa with higher prevalence of *Plasmodium falciparum* (in Guinea). The *Rsb* index here was obtained using 9521 SNPs with minor allele frequency >5% across the two populations, with deviation to the left hand side for SNPs having a stronger integrated haplotype score in Mauritania in comparison with Guinea, and to the right indicating stronger scores in Guinea. There was no evidence of selection that was stronger in Mauritania relative to Guinea but five signatures specific to Guinea were detected (labelled a–e, Table S9): (a) chromosome (chr) 2 position 100.4–100.6 kb covering PF3D7_020200 and supported by 3 SNPs, (b) chr 6 position 1115.5–1141.1 kb covering 4 genes (PF3D7_0627900‐PF3D7_0628100 and supported by 10 SNPs, (c) chr 6 position 1252.1–1266.6 kb covering 4 genes (PF3D7_0630000‐PF3D7_0630300 and supported by 7 SNPs, (d) chr 9 position 88.4–88.6 kb covering no genes and supported by 2 SNPs, (e) chr 9 position 1179.0–1190.4 kb covering 7 genes (PF3D7_0929400‐PF3D7_0930000 and supported by 5 SNPs).

Finally, to explore whether there were genomic regions with population‐specific signatures of selection indicated by allele‐specific extended haplotype homozygosity at particular loci, a cross‐population comparison was conducted using the data from Mauritania and Guinea. The *Rsb* index provides a contrast between populations in the extent of haplotype homozygosity for each SNP allele compared with its alternative, contrasting the average haplotype length in one population relative to that in the second population. Using a cut‐off of at least 2 SNPs with |*Rsb*| values >5, no genomic regions were detected with signatures that were stronger in Mauritania than in Guinea, but five regions had signatures that were stronger in Guinea (Fig. 8). The strongest two of these were both near the end of chromosome 6, and overlap with the region of high |iHS| values shown above for the Mauritanian sample, for which high |iHS| values had also previously been shown for the Guinea population sample (Mobegi *et al*. 2014). Inspection of the SNP genotype profiles in this chromosome 6 region indicates that haplotypes at elevated frequency in both populations are related. The *Rsb* result implies that, although haplotype lengths in both populations are longer in this region relative to the genome as a whole, the relative length in Guinea is longer than that in Mauritania, suggesting that selection has been stronger or more recent in Guinea. The other three regions with |*Rsb*| values indicating stronger selection in Guinea, one in chromosome 2 and two in chromosome 9 (Fig. 8 and Table S9, Supporting information), do not exhibit any indices of selection in the Mauritanian population sample.

## Discussion

This study characterizes the genetic structure and signatures of selection in populations of malaria parasites living in an exceptional environment, at the limit of the African endemic distribution which approaches the edge of the Sahara desert. An immediately apparent feature was that the numbers of genotypes per infection were lower than has been seen elsewhere within West Africa, although the overall allelic diversity was similar (Mobegi *et al*. 2012; Oyebola *et al*. 2014). This is to be expected as transmission by mosquitoes is relatively rare in these arid areas with limited seasonal rainfall, which reduces the occurrence of superinfection by different genotypes. Although most infections had unrelated parasite genotypes, a minority shared identical or closely related genotypes, indicating that reduced opportunity for outcrossing has led to self‐fertilization and sporadic expansion of genetically identical parasite clonal lineages. Such a population structure may be characterized as indicating an ‘epidemic’ situation (Maynard Smith *et al*. 1993; Anderson *et al*. 2000), in which the rate of infection transmission is intermittent and unstable, yet it is common enough to prevent the parasite population from becoming predominantly clonal which would be expected in populations approaching the point at which they may be locally eliminated (Nkhoma *et al*. 2013).

The occurrence of small clusters of closely related or identical genotypes was responsible for the significant multilocus linkage disequilibrium indices seen at four of eight endemic locations, and also caused moderately significant allele frequency divergence at two of the sampled sites. Aside from these few local genotype clusters, the underlying parasite populations had similar genotypic profiles despite being sampled from diverse sites over a range of ~1000 km across the *Plasmodium falciparum* endemic area of the country. This indicates that apart from occasional local epidemic expansion of genotypes in local *P. falciparum* populations in Mauritania, there is ongoing recombination and gene flow, so that the populations are not genetically fragmented enough to identify isolated foci of infection that could be feasibility eliminated in the immediate future. This is an important finding, as there are increasing international expectations that Mauritania may be one of the countries in Africa that should aim to achieve malaria elimination (Newby *et al*. 2016). A key process that needs to be studied quantitatively is human population movement, about which data are very limited in this region, although increasing efforts to study the issue are being made for countries in southern Africa on the opposite edge of the geographical distribution of malaria endemicity (Ruktanonchai *et al*. 2016). Although migration may also be a feature of mosquito vector populations colonizing new breeding sites after the seasonal rains begin, as indicated in ecological studies elsewhere in the Sahel (Dao *et al*. 2014), it is unlikely that vectors transport parasites as efficiently as humans over large distances.

In the overall population sample of sequences from Mauritania, several regions of the *P. falciparum* genome showed evidence of selection as indicated by standardized integrated haplotype scores, with four of the strongest signatures overlapping with or in close proximity to antimalarial drug resistance genes (the chloroquine resistance genes *crt* and *mdr1*, as well as the antifolate resistance genes *dhfr* and *dhps*). The overall summary of the genomewide allele frequency spectrum as indicated by the negative Tajima's *D* index in Mauritania was similar to that previously seen in a more highly endemic population elsewhere in the West African region, but it is notable that a few particular genomic loci showed marked differences in allele frequency. Most of the SNPs with highly divergent frequencies mapped within or adjacent to the antimalarial drug resistance genes *crt*,* mdr1*, and *dhps*, known to have spatial and temporal allele frequency variation elsewhere in West Africa due to historical drug selection (Nwakanma *et al*. 2014). The remaining highly divergent frequency SNP is within gene *Rh1* which encodes one of several parasite ligands that bind to alternative receptors for erythrocyte invasion (Wright & Rayner 2014), and it is not yet known whether the particular nonsynonymous change at codon 191 or another linked polymorphism has an adaptive effect. Interestingly, given marked difference in levels and seasonality of transmission, no allele frequency differences were seen at any locus known to be involved in development of parasite transmission stages. A previous contrast of a low transmission area in the coastal part of The Gambia with the highly endemic population from Guinea showed the *gdv1* (gametocyte development protein 1) gene locus to have the most highly divergent SNP allele frequencies (Mobegi *et al*. 2014), so the lack of divergence at this locus between Mauritania and Guinea suggests that selection is not simply related to the amount of local transmission. This illustrates the need for multiple population studies, sampling across different environments with a broad range of epidemiological and ecological variation.

The range of malaria parasite endemicity is subject to changes in environment, which will potentially expand the global distribution range in particular directions and cause it to contract elsewhere (Rogers & Randolph 2000; Gething *et al*. 2010). The northern edge of the distribution of *P. falciparum* in Africa is principally determined by very limited rainfall which restricts the ability of vector mosquitoes, to breed and transmit infection during a short annual season. However, this is not the absolute limit for malaria, as it has recently been discovered that another species of human malaria parasite, *Plasmodium vivax*, persists further north in Mauritania (Ba *et al*. 2016). This is a distantly related malaria parasite species that can persist in human communities where mosquito transmission occurs even more rarely, as the parasite has a dormant stage in the liver which leads to relapses and maintenance of endemicity over many years. Where they occur together elsewhere, it is generally seen that *Plasmodium falciparum* decreases more rapidly than *P. vivax* in response to malaria control (WHO, 2015). Although enhanced control efforts at the edges of *P. falciparum* distribution in Africa are required from a public health perspective, this study indicates that migration from more central parts of its endemic range will make it very difficult to achieve local elimination.

Although many species face extreme environments on the edges of their geographic range which limit the fitness of local populations (Gaston 2003), it is rarely known whether local adaptive potential is constrained, either by genetic drift due to lower effective population sizes or by inflow of genes from more highly populated areas towards the centre of the species range (Bridle & Vines 2007; Eckert *et al*. 2008). Reduced adaptability at range edges might predict feasibility of eliminating particular populations of pest or pathogen species (Shapiro & Polz 2014), but to establish if this is the case for malaria parasites would require analysis of multiple edge areas. In Africa, this should involve detailed analysis of other rarely‐studied areas, in the north‐eastern edge of the endemic distribution, as well as in the south.

In Mauritania, it is clear that the low transmission Sahel environment has had limited impact upon the parasite population structure, reducing numbers of genotypes per infection compared to the rest of West Africa, although overall local levels of allelic diversity were not lower. A minority of infections contained identical or highly related genotypes within a few of the locations, causing slight effects on multilocus linkage disequilibrium and divergence of allele frequencies, but otherwise there was minimal divergence between locations. Analysis of genomewide data indicates that positive directional selection has affected multiple loci, and comparison with data from a more highly endemic area of West Africa highlights several loci with allele frequency divergence, but does not identify any loci to be only under selection in Mauritania. From an immediate applied perspective, the results show that the parasite populations are not significantly fragmented genetically, and suggest that unprecedented efforts would be required to sustainably eliminate malaria from the northern edge of its range in Africa.

H.B. and D.J.C. conceived, designed and oversaw the study. C.W.D., S.A., A.D.A., B.D.T., A.T. and F.K. collected the samples and performed laboratory assays. D.P.K. organized the process of genome sequencing, bioinformatic SNP calling through the MalariaGEN pipeline, and nucleotide data deposition. C.W.D., S.A., F.K. and D.J.C. performed data analysis and interpretation. C.W.D. and D.J.C. wrote the manuscript. All authors read and approved the final manuscript.

## Data accessibility

Microsatellite genotype data for each of the 203 individual *Plasmodium falciparum* infections analysed are given in full in Table S1 (Supporting information). Genome sequence data for each of the 65 individual *P. falciparum* infections analysed are freely accessible through the European Nucleotide Archive, as listed in Table S4 (Supporting information). All SNP genotype calls together with guidelines for data use are also given in an openly accessible project page on the MalariaGEN site https://www.malariagen.net/resource/22.

## Supporting information


**Fig. S1** Principal co‐ordinates analysis (PCoA) of variation among 10‐locus microsatellite genotypes of *P. falciparum* clinical isolates sampled from eight different locations in Mauritania in 2012–2013.
**Fig. S2** Principal components analysis (PCA) of variation among genome‐wide SNP profiles of *P. falciparum* clinical isolates sampled from four different locations in Mauritania in 2014.
**Fig. S3** Principal components analysis (PCA) of variation among genome‐wide SNP profiles of *P. falciparum* clinical isolates sampled from Mauritania (current study) and a previous population sample from the Republic of Guinea (Mobegi *et al*. 2014; *Mol. Biol. Evol*. 31:1490–99).
**Fig. S4** Genome‐wide scan of Jost's *D*est index of SNP frequency differentiation between *P. falciparum* from Mauritania (current study) and a previous population sample from the Republic of Guinea (Mobegi *et al*. 2014; *Mol. Biol. Evol*. 31:1490–99).
**Table S1** Microsatellite genotypes for 203 *P. falciparum* clinical infections sampled from eight different locations in Mauritania.
**Table S2** Allelic diversity (expected heterozygosity, *H*_e_) of *P. falciparum* at 10 microsatellite loci at eight endemic locations in Mauritania (sample sizes are given in the paper).
**Table S3** Estimates of effective *P. falciparum* population size (*N*_e_) based on the observed local microsatellite allele diversity (*H*_e_) assuming a standard mutation rate under either a stepwise mutation model (SMM) or an infinite alleles model (IAM), at each of the Mauritanian sites with sample sizes of at least 10 infections and at other sites in four previously studied West African countries.
**Table S4** Pairwise values estimating differentiation between local populations in Mauritania summarising data for 10 microsatellite loci as measured with a) *F*_ST_ and b) Jost's *D*est.
**Table S5** Sequence accession numbers, intra‐infection SNP frequency fixation indices (*F*_WS_) and pairwise differences among individual *P. falciparum* clinical infection samples from Mauritania.
**Table S6** Overall population Tajima's *D* values for genes with at least 3 SNPs, based on allele frequency distributions of the majority allele called in each of 65 *P. falciparum* clinical infection samples from Mauritania.
**Table S7** Windows of the *P. falciparum* genome containing elevated standardised integrated haplotype scores in the overall analysis of genomewide SNP diversity in Mauritania.
**Table S8.** Genomic positions of *P. falciparum* SNPs with *F*_ST_ values >0.2 comparing the overall Mauritanian population sample with a previously published population sample from a more highly endemic area in the Republic of Guinea (Mobegi *et al*. 2014, *Mol. Biol. Evol*. 31:1490–99).
**Table S9** Windows across the *P. falciparum* genome for which extended haplotypes were observed in Guinea relative to Mauritania as detected by *Rsb* analysis. No windows of extended haplotype in Mauritania relative to Guinea were detected from this scan.
