# Supplementary material for: Population genetic structure and adaptation of malaria parasites on the edge of endemic distribution
Source: Mol Ecol. 2017 Mar 15;26(11):2880–94. doi: 10.1111/mec.14066 (PMC5485074; doi:10.1111/mec.14066)

Supporting Information

Population genetic structure and adaptation of malaria parasites on the edge of endemic distribution

Craig W. Duffy [1*], Hampate Ba [2], Samuel Assefa [1], Ambroise D. Ahouidi [3], Yacine B. Deh [2], Abderahmane Tandia [2], Freja C.M. Kirsebom [1], Dominic P. Kwiatkowski [4], David J. Conway [1*]

[1] Department of Pathogen Molecular Biology, London School of Hygiene & Tropical Medicine, London, Keppel St, UK

[2] Institut National de Recherche en Sante Publique, Nouakchott, Mauritania

[3] Laboratory of Bacteriology and Virology, Le Dantec Hospital, Cheikh Anta Diop University, Dakar, Senegal

[4] Malaria Programme, Wellcome Trust Sanger Institute, Hinxton, UK

Supporting Information for this paper consists of nine tables (**Tables S1 – S9**), and four figures (**Figures S1 – S4**).

This pdf document contains all of the tables and figures, except for three tables that are given separately as EXCEL files (**Tables, S1, S5 and S6**)

Table S1. Microsatellite genotypes for 203 *P. falciparum* clinical infections sampled from eight different locations in Mauritania. (This is an EXCEL spreadsheet database that is given separately as it is large and will not combine into a readable pdf file).

Table S2. Allelic diversity (expected heterozygosity, H_e) of *P. falciparum* at 10 microsatellite loci at eight endemic locations in Mauritania (sample sizes are given in the paper).

	Allelic diversity (expected heterozygosity, H_e) at 10 microsatellite loci										
	ta87	*ta109*	*ta42*	*polya*	*ta60*	*ta81*	*ara2*	*pfg377*	*pfpk2*	*ta1*	Mean
Location											
Aioun	0.833	0.892	0.000	0.917	0.842	0.767	0.783	0.492	0.817	0.908	0.725
Kiffa	0.952	0.857	0.524	0.905	0.810	0.857	0.952	0.667	0.905	0.714	0.814
Kobeni	0.869	0.854	0.415	0.880	0.801	0.778	0.845	0.523	0.866	0.886	0.772
Nema	0.890	0.832	0.416	0.890	0.868	0.779	0.853	0.416	0.737	0.832	0.751
Nouakchott	0.882	0.837	0.314	0.915	0.699	0.719	0.843	0.706	0.811	0.882	0.761
Ould Yenge	0.873	0.855	0.436	0.927	0.836	0.836	0.600	0.618	0.873	0.855	0.771
Selibaby	0.887	0.836	0.091	0.840	0.805	0.801	0.801	0.558	0.801	0.857	0.728
Timbedra	0.858	0.767	0.233	0.917	0.808	0.867	0.883	0.542	0.925	0.858	0.766
Total number of alleles	12	16	10	18	10	12	11	7	16	15	12.7

Table S3. Estimates of effective *P. falciparum* population size (N_e) based on the observed local microsatellite allele diversity (H_e) assuming a standard mutation rate under either a stepwise mutation model (SMM) or an infinite alleles model (IAM), at each of the Mauritanian sites with sample sizes of at least 10 infections (specified in the paper) and at other sites in four previously studied West African countries.

Site sampled (*and country*)		SMM N_e (95% C.I.)	IAM N_e (95% C.I.)
Mauritania	Aioun	9560 (4129-21890)	4124 (1781-9443)
	Kobeni	14264 (6161-32651)	5297 (2288-12127)
	Nema	11833 (5111-27093)	4718 (2038-10803)
	Nouakchott	12911 (5577-29561)	4981 (2151-11404)
	Ould Yenge	14133 (6104-32359)	5267 (2275-12059)
	Selibaby	9790 (4229-22415)	4189 (1808-9586)
	Timbedra	13503 (5832-30915)	5121 (2212-11725)
Senegal	Richard Toll	9195 (3971-21051)	4023 (1737-9210)
The Gambia	Greater Banjul	11733 (5068-26862)	4693 (2027-10745)
	Farafenni	11733(5068-26862)	5693 (2027-10745)
	Basse	12797 (5527-29300)	4954 (2140-11342)
Guinea Bissau	Caio	18772 (8108-42980)	6257 (2703-14327)
Guinea	Forecariah	14004 (6049-32062)	5237 (2262-14327)
	Boke	16954 (7323-38818)	5885 (2542-13474)
	N'Zerekore	15379 (6642-35210)	5546 (2396-12699)

Table S4. Pairwise values estimating differentiation between local populations in Mauritania summarising data for 10 microsatellite loci as measured with a) F_{ST} and b) Jost's D_{est}.

a) F_{ST}

	Aioun	Selibaby	Kobeni	Nouakchott	Nema	Ould Yenge	Timbedra
Aioun		0.030*	0.008	0.024*	0.019*	0.048*	0.004
Selibaby	0.007		0.020**	0.005	0.027*	0.036**	0.025**
Kobeni	0.002	0.000		0.003	0.013	0.026	0.007
Nouakchott	0.018	0.000	0.005		0.018	0.005	0.012
Nema	0.016*	0.013	0.009	0.019		0.013	0.002
Ould Yenge	0.029	0.016	0.014	0.000	0.011		0.027
Timbedra	0.002	0.007	0.009	0.012	0.000	0.015	

b) Jost's D_{est}

	Aioun	Selibaby	Kobeni	Nouakchott	Nema	Ould Yenge	Timbedra
Aioun	-	0.075*	0.025	0.085*	0.053	0.139**	0.012
Selibaby	0.014	-	0.060**	0.029	0.069*	0.103*	0.066
Kobeni	0.008	0.004	-	0.014	0.043*	0.085**	0.025
Nouakchott	0.068*	< 0.001	0.014	-	0.077*	0.014	0.056
Nema	0.044	0.025	0.036	0.078*	-	0.039	0.005
Ould Yenge	0.107*	0.073	0.067*	0.019	0.030	-	0.088*
Timbedra	0.006	0.011	0.026	0.056	< 0.001	0.074	-

Top right matrices show analysis based on all infection samples; bottom left matrices show analysis based on samples with unique genotypes. Pairwise comparisons with values significantly different from zero are asterisked (* $P < 0.05$, ** $P < 0.01$).

Table S5. Sequence accession numbers, intra-infection SNP frequency fixation indices (F_{WS}) and pairwise differences among individual *P. falciparum* clinical infection samples from Mauritania. (This is an EXCEL spreadsheet database that is given separately).

Table S6. Overall population Tajima's D values for genes with at least 3 SNPs, based on allele frequency distributions of the majority allele called in each of 65 *P. falciparum* clinical infection samples from Mauritania. (This is an EXCEL spreadsheet database that is given separately as it is large and will not combine into a readable pdf file).

Table S7. Windows of the *P. falciparum* genome containing elevated standardised integrated haplotype scores in the overall analysis of genome-wide SNP diversity in Mauritania.

Region	Chromosome	Number of SNPs with |iHS| > 3.29 *	Number of SNPs with |iHS| > 5 **	Left boundary position (Kb)	Right boundary position (Kb)	Window Size (Kb)
a	4	7	1	673	765	92
b	5	5	2	908	1000	91
c	6	37	20	1087	1271	183
d	7	41	8	196	701	504
e	8	3	1	486	506	19
f	8	3	1	626	703	77

* SNPs with |iHS| > 3.29 are in the top 0.1% of genome-wide values.

Table S8. Genomic positions of *P. falciparum* SNPs with F_{ST} values > 0.2 comparing the overall Mauritanian population sample with a previously published population sample from a more highly endemic area in the Republic of Guinea (Mobegi *et al.* 2014, *Mol. Biol. Evol.* 31:1490-99)

Chromosome	Position	Gene	Codon change	F_{ST}	Reference allele frequency Mauritania / Guinea	Distance to nearest drug resistance gene
4	138308	RH1, PF3D7_0402300	E191K	0.2258	0.62 / 0.26	609.7kb
5	966295	Unknown function, PF3D7_0523200	F58S	0.2227	0.68 / 0.94	4.1kb
7	409108	CG1, PF3D7_0709100	Synonymous, codon 298	0.3242	0.88 / 0.44	2.8kb
7	409122	CG1, PF3D7_0709100	S303N	0.3242	0.88 / 0.44	2.8kb
7	409168	CG1, PF3D7_0709100	I318M	0.2722	0.86 / 0.47	2.9kb
7	415245	CG2, PF3D7_0709300	H2169Y	0.2022	0.25 / 0.59	8.9kb
7	415250	CG2, PF3D7_0709300	N2167S	0.2022	0.25 / 0.59	8.9kb
8	544529	RNA helicase, PF3D7_0810600	H853Q	0.2069	0.80 / 0.45	3.7kb
8	549685	DHPS, PF3D7_0810800	G437A	0.2036	0.37 / 0.71	0

Table S9. Windows across the *P. falciparum* genome for which extended haplotypes were observed in Guinea relative to Mauritania as detected by *Rsb* analysis. No windows of extended haplotype in Mauritania relative to Guinea were detected from this scan.

Chromosome	Number of SNPs with Rsb < -5	Window size, kb (Start-End Position)	Genes within region (Number)
2	3	5.8 (100.4-106.2)	PF3D7_020200 (1)
6	10	25.6 (1115.5-1141.1)	PF3D7_0627800 – PF3D7_0628100 (4)
6	7	14.5 (1252.1-1266.6)	PF3D7_0630000 – PF3D7_0630300 (4)
9	2	0.2 (88.4-88.6)	- (0)
9	5	11.4 (1179.0-1190.4)	PF3D7_0929400 – PF3D7_0930000 (7)

Figure S1. Principal co-ordinates analysis (PCoA) of variation among 10-locus microsatellite genotypes of *P. falciparum* clinical isolates sampled from eight different locations in Mauritania in 2012-2013. The top panel colours the data by sample year, and the bottom panel by sample location.

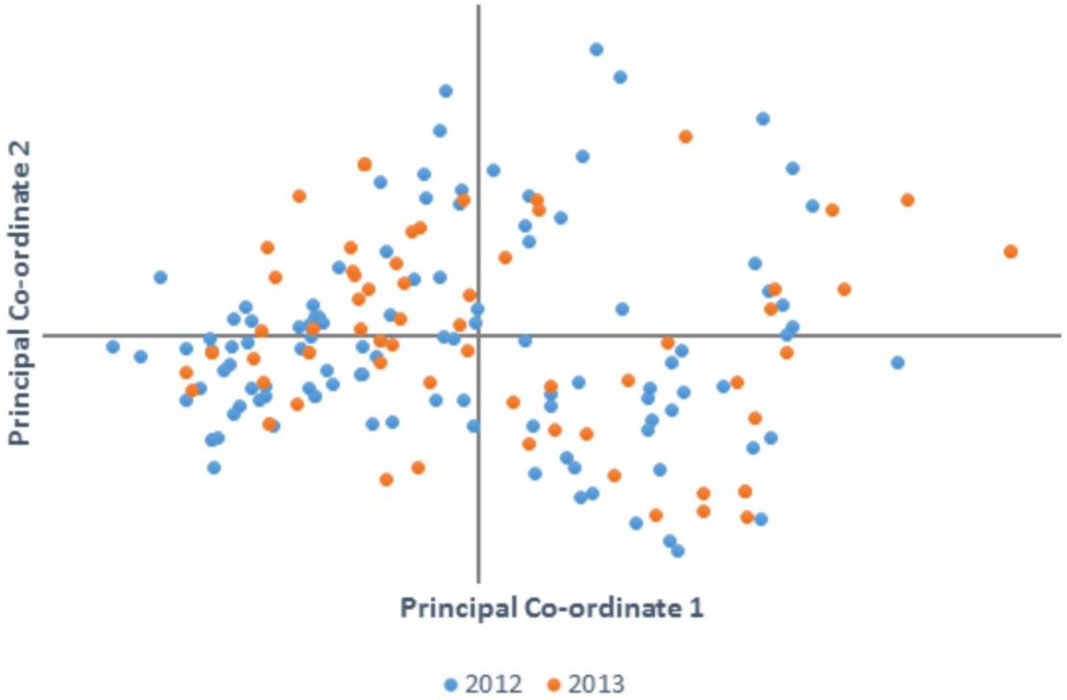

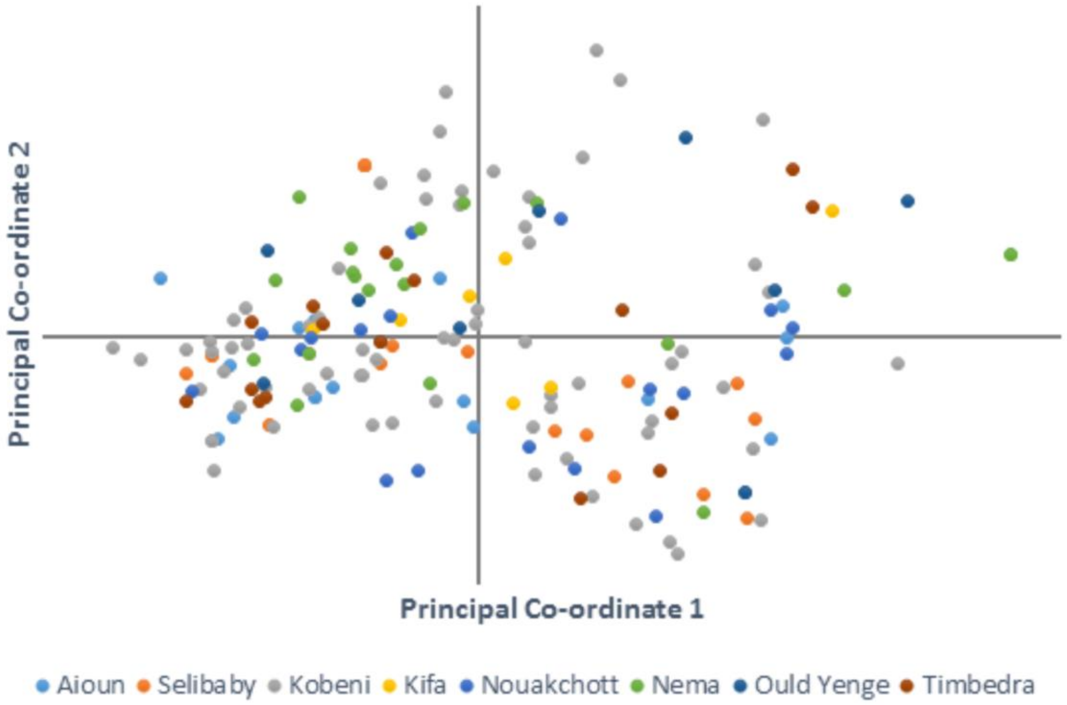

Figure S2. Principal components analysis (PCA) of variation among genome-wide SNP profiles of *P. falciparum* clinical isolates sampled from four different locations in Mauritania in 2014.

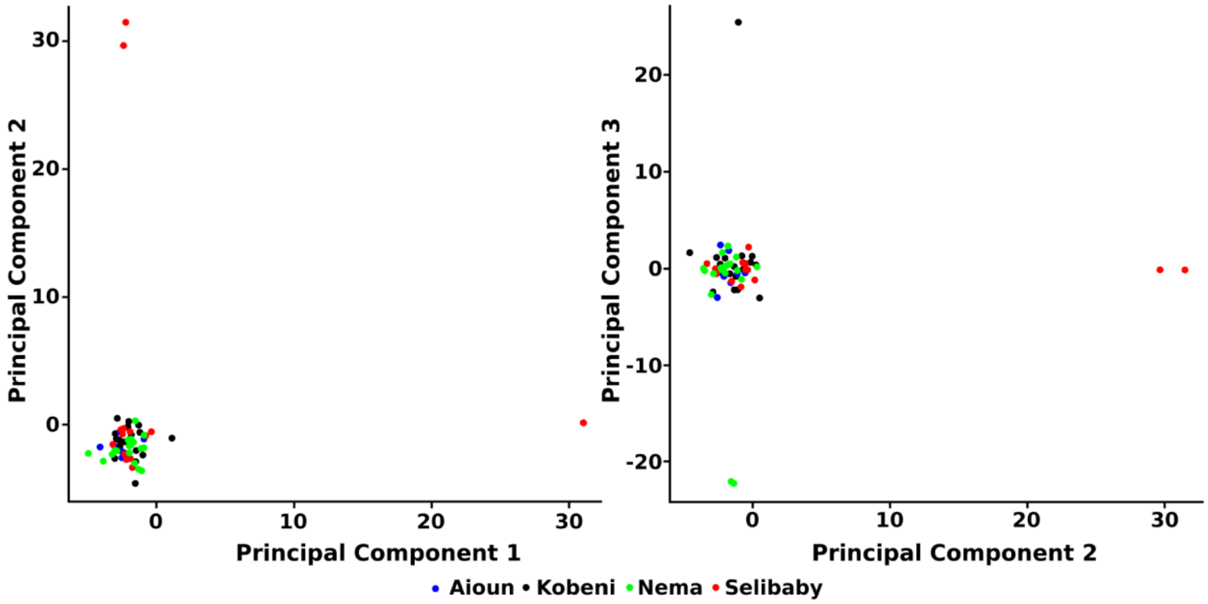

Figure S3. Principal components analysis (PCA) of variation among genome-wide SNP profiles of *P. falciparum* clinical isolates sampled from Mauritania (current study) and a previous population sample from the Republic of Guinea (Mobegi *et al.* 2014, *Mol. Biol. Evol.* 31:1490-99).

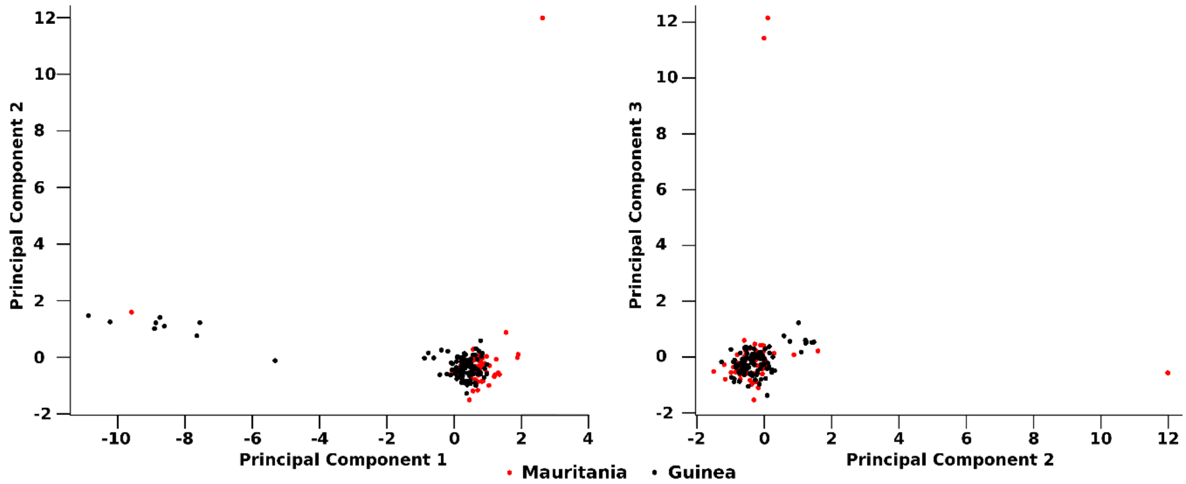

Figure S4. Genome-wide scan of Jost's D_{est} index of SNP frequency differentiation between *P. falciparum* from Mauritania (current study) and a previous population sample from the Republic of Guinea (Mobegi *et al.* 2014, *Mol. Biol. Evol.* 31:1490-99). Arrows identify four loci as labelled also on Figure 7.

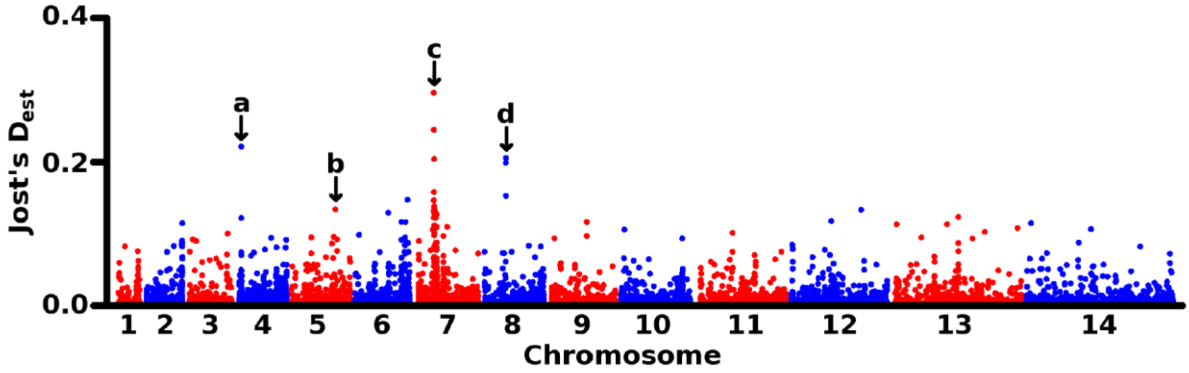

Supplement: Supplementary file 1 — Fig. S1 Principal co‐ordinates analysis (PCoA) of variation among 10‐locus microsatellite genotypes of P. falciparum clinical isolates sampled from eight different locations in Mauritania in 2012–2013. Fig. S2 Principal components analysis (PCA) of variation among genome‐wide SNP profiles of P. falciparum clinical isolates sampled from four different locations in Mauritania in 2014. Fig. S3 Principal components analysis (PCA) of variation among genome‐wide SNP profiles of P. falciparum clinical isolates sampled from Mauritania (current study) and a previous population sample from the Republic of Guinea (Mobegi et al. 2014; Mol. Biol. Evol. 31:1490–99). Fig. S4 Genome‐wide scan of Jost's Dest index of SNP frequency differentiation between P. falciparum from Mauritania (current study) and a previous population sample from the Republic of Guinea (Mobegi et al. 2014; Mol. Biol. Evol. 31:1490–99). Table S1 Microsatellite genotypes for 203 P. falciparum clinical infections sampled from eight different locations in Mauritania. Table S2 Allelic diversity (expected heterozygosity, H e) of P. falciparum at 10 microsatellite loci at eight endemic locations in Mauritania (sample sizes are given in the paper). Table S3 Estimates of effective P. falciparum population size (N e) based on the observed local microsatellite allele diversity (H e) assuming a standard mutation rate under either a stepwise mutation model (SMM) or an infinite alleles model (IAM), at each of the Mauritanian sites with sample sizes of at least 10 infections and at other sites in four previously studied West African countries. Table S4 Pairwise values estimating differentiation between local populations in Mauritania summarising data for 10 microsatellite loci as measured with a) F ST and b) Jost's Dest. Table S5 Sequence accession numbers, intra‐infection SNP frequency fixation indices (F WS) and pairwise differences among individual P. falciparum clinical infection samples from Mauritania. Table S6 Overal [file MEC-26-2880-s001.pdf]
